# Finding a cure for HIV: will it ever be achievable?

**DOI:** 10.1186/1758-2652-14-4

**Published:** 2011-01-24

**Authors:** Sharon R Lewin, Vanessa A Evans, Julian H Elliott, Bruno Spire, Nicolas Chomont

**Affiliations:** 1 Department of Medicine, Monash University, (99 Commercial Rd), Melbourne, (3004), Australia; 2 Infectious Diseases Unit, Alfred Hospital, (85 Commercial Rd), Melbourne, (3004), Australia; 3 Centre of Virology, Burnet Institute, (85 Commercial Rd), Melbourne, (3004), Australia; 4 SE4S, INSERM UMR 912, (23 rue Stanislas Torrents), Marseille, (13006), France; 5 SE4S, Université de la Méditerranée, IRD, (23 rue Stanislas Torrents), Marseille, (13006), France; 6 AIDES, (14 rue Scandicci, Pantin (93508), France; 7 Vaccine and Gene Therapy Institute, (11350 SW Village Parkway), Port St Lucie, (34987), FL, USA

## Abstract

Combination antiretroviral therapy (cART) has led to a major reduction in HIV-related mortality and morbidity. However, HIV still cannot be cured. With the absence of an effective prophylactic or therapeutic vaccine, increasing numbers of infected people, emerging new toxicities secondary to cART and the need for life-long treatment, there is now a real urgency to find a cure for HIV.

There are currently multiple barriers to curing HIV. The most significant barrier is the establishment of a latent or "silent" infection in resting CD4+ T cells. In latent HIV infection, the virus is able to integrate into the host cell genome, but does not proceed to active replication. As a consequence, antiviral agents, as well as the immune system, are unable to eliminate these long-lived, latently infected cells. Reactivation of latently infected resting CD4+ T cells can then re-establish infection once cART is stopped. Other significant barriers to cure include residual viral replication in patients receiving cART, even when the virus is not detectable by conventional assays. In addition, HIV can be sequestered in anatomical reservoirs, such as the brain, gastrointestinal tract and genitourinary tract.

Achieving either a functional cure (long-term control of HIV in the absence of cART) or a sterilizing cure (elimination of all HIV-infected cells) remains a major challenge. Several studies have now demonstrated that treatment intensification appears to have little impact on latent reservoirs. Some potential and promising approaches that may reduce the latent reservoir include very early initiation of cART and the use of agents that could potentially reverse latent infection.

Agents that reverse latent infection will promote viral production; however, simultaneous administration of cART will prevent subsequent rounds of viral replication. Such drugs as histone deacetylase inhibitors, currently used and licensed for the treatment of some cancers, or activating latently infected resting cells with cytokines, such as IL-7 or prostratin, show promising results in reversing latency *in vitro *when used either alone or in combination. In order to move forward toward clinical trials that target eradication, there needs to be careful consideration of the risks and benefits of these approaches, agreement on the most informative endpoints for eradication studies and greater engagement of the infected community.

## Introduction

The XI International AIDS Conference in Vancouver in 1996 marked the beginning of the great success story of combination antiretroviral therapy (cART). Over the past 15 years, mortality and morbidity from HIV has fallen dramatically in both resource-poor and resource-rich countries [1-3]. Treatment has become simpler and less toxic, and more than 5 million people in low- and middle-income countries are now receiving cART [4]. Despite these major successes, and in the absence of an effective vaccine, the need to find a cure for HIV is even more urgent now, in 2010, than ever before.

## Discussion

### Why do we need a cure for HIV?

Even with the major successes of cART, full life expectancy for patients living with HIV has not been restored. In a prospective study of 3990 HIV-infected individuals and 379,872 HIV-uninfected controls in Denmark, the probability of survival was examined in the period prior to cART (1995-1996), during early cART (1997-1999) and during late cART (2000-2005) [5]. There was a clear and substantial increase in survival following the introduction of cART in the late 1990s. However, even in the late cART period, life expectancy remained significantly less than population controls. In fact, the chance of a person with HIV reaching the age of 70 was 50% that of uninfected population controls. These findings are consistent with observations from other large cohort studies [6].

The incidence of significant morbidity remains elevated despite successful cART due to complex interactions between drug toxicity [7], persistent inflammation [8] and risk behaviours [9]. Multiple studies have demonstrated that people living with HIV are at increased risk of cardiovascular disease, metabolic disorders, neurocognitive abnormalities, liver and renal disease, bone disorders, malignancy and frailty (reviewed in [10]). As a consequence, managing the complex care needs of HIV-infected individuals remains a major challenge.

Finally, despite the clear need for universal access to cART and the ongoing expansion in health systems, there remains a lack of financial resources to support life-long treatment, for everyone in need of treatment. Reaching all those in need of treatment is getting harder as donor contributions stabilize and treatment recommendations shift towards earlier initiation of cART [11,12], which will increase the population of people judged to be in need of treatment. Furthermore, new HIV infections continue to outpace the number of people starting treatment. Even during the rapid scale up of access to cART in recent years, for every two people starting cART, there were five new infections [13]. This imbalance is unlikely to be reversed in the near future despite evidence that global HIV incidence is now declining [14] and the promise of more effective biomedical interventions, including circumcision and tenofovir-containing microbicides [15,16].

Recent work, commissioned by the Clinton Foundation as part of the AIDS 2031 Project, has modelled the total projected annual AIDS resource requirements for low-and middle-income countries if cART scale up continues at current rates [17]. If HIV treatment is initiated at a CD4 count of 200 cells/mm^3 ^and 40% cART coverage is achieved, the estimated costs by 2031 are predicted to approach $25 billion per year. If cART coverage instead reaches 80% by 2031, the annual cost of treatment is predicted to reach almost $35 billion [17]. Under this scenario, which is broadly consistent with the international community's commitment to universal access, the predicted cost of HIV treatment alone will account for almost half the US foreign aid budget by 2016 [13].

### Current barriers to curing HIV

Following cART, HIV RNA in blood rapidly reduces to undetectable levels (<50 copies/ml). However, regardless of whether the patient has been on treatment for two years or 15 years, whether they have been on three drugs or six drugs, whether they started treatment within one year or 10 years of infection, as soon as treatment is stopped, the virus rapidly rebounds. The question then is: where is the virus sitting while the patient has a viral load of less than 50 copies/ml?

More than 10 years ago, several groups identified the persistence of virus in long-lived latently infected cells, measured as HIV DNA. They demonstrated that upon stimulation, these silent viral genomes can be reactivated and subsequently produce infectious viral particles [18-20]. More recently, using a highly sensitive assay that detects HIV RNA in plasma down to 1 copy/ml, several groups have shown persistent low-level viremia of around 3-5 copies/ml in 80% of patients [21,22]. In other words, there is no such thing as an undetectable viral load and the virus clearly persists. Currently, some of the major research questions are: what is contributing to this low-level viremia and persistent DNA, and will it ever be possible to eliminate this residual virus?

There are likely to be at least three major barriers to curing HIV. These include the persistence of long-lived, latently infected cells, residual viral replication and anatomical reservoirs. Latently infected cells are predominantly resting CD4+ T cells [18-20], but also include other long-lived cells, such as monocyte/macrophages [23] and astrocytes [24,25]. Latency represents the biggest challenge to finding a cure.

*In vivo*, HIV latency occurs in resting CD4+ T cells either as pre-integration or post-integration latency. Pre-integration latency refers to unintegrated HIV DNA that is unstable and will either degrade or will integrate into the host cell genome, usually following cell activation [26]. Post-integration latency refers to the presence of integrated HIV DNA in cells that are not actively producing viral particles. The major reservoir of cells that harbour post-integration latency *in vivo *are resting memory CD4+ T cells [27,28]. Once integration occurs, the virus can persist in these cells for long periods of time, unaffected by antiretroviral drugs or host immune recognition [19,29]. Post-integration latency is therefore critical for the maintenance of the HIV latent reservoir.

In activated CD4+ T cells, the virus life cycle is efficient, with rapid integration, virion production and subsequent death of the infected cells. In contrast, infection of resting CD4+ T cells is difficult to establish *in vitro *due to multiple blocks in the viral life cycle [30,31]. However, resting CD4+ T cells are clearly infected *in vivo *[32,33], as well as *ex vivo*, in tissue blocks [34,35], and contain stable integrated forms of HIV.

**Figure 1:**
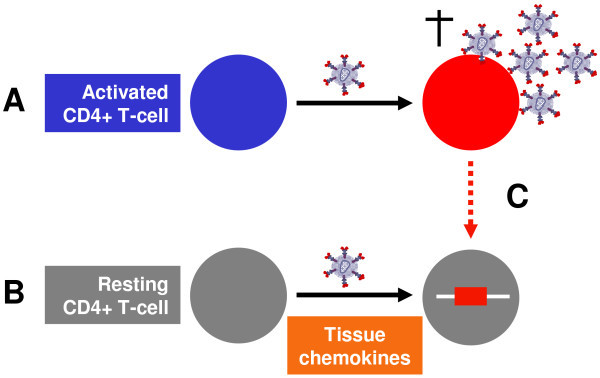
**HIV latency and infection of resting memory CD4+ T cells**. **(A) **In activated CD4+ T cells the virus life cycle is efficient, with rapid integration, virion production and subsequent death of the infected cells. **(B) **Latent infection can be established in CD4+ resting memory T cells following incubation with multiple chemokines [36,37]. **(C) **Alternatively, latently infected cells may arise following the reversion of an infected activated cell to a resting state [38-40].

*In vitro*, our group has clearly demonstrated that latent infection can be established in CD4+ resting memory T cells, following incubation with multiple chemokines that bind to the chemokine receptors highly expressed on resting CD4+ T cells [36,37]. Studies such as this support the hypothesis that latency can result from direct infection of resting memory CD4+ T cells, possibly as a result of exposure to soluble factors found in lymphoid tissues. An alternative possibility for infection of resting CD4+ T cells is the reversion of an infected, activated cell to a resting state, which has also been demonstrated *in vitro *[38-40] (Figure 1).

These latently infected CD4+ T cells are thought to be extremely long lived [41]; however, it is highly likely that this pool of cells is also maintained by homeostatic proliferation [27]. Latently infected cells can intermittently release virus following activation, making them a major barrier to cure. Latency was originally described in one particular subset of CD4+ T cells called central memory T cells. Because these cells can persist for decades, they ensure the maintenance of long-lasting cellular immunity, but also constitute an extremely stable cellular reservoir for the virus [18,19].

Over the past few years, several groups have identified that latency can exist in a range of CD4+ T cell subsets, including transitional memory T cells, naïve T cells, thymocytes and multipotent progenitor cells or stem cells [27,42-44]. Together, these cells constitute the latent reservoir. There is also some indirect evidence that the normal process of homeostatic proliferation maintains the number of latently infected naïve and transitional memory cells. Following mitotic cell division, both daughter cells contain integrated HIV DNA, meaning that this reservoir may be replenished or even increase in size on cART [27,44].

The alternative explanation for persistent virus is that there is ongoing virus replication in activated T cells. In other words, cART is effective, but not 100% effective. However, it is currently unclear how much residual replication contributes to HIV persistence. There are several pieces of evidence that argue against residual replication, including the very stable sequence of low-level viremia in plasma [45,46] and the absence of drug-resistant virus in either plasma or CD4+ T cells [47,48].

Finally, we know that HIV can hide in anatomical reservoirs, such as the brain [49], the gastrointestinal tract [50] and the genital tract [51]. In the gastrointestinal tract of patients receiving cART, persisting infected cells are almost 10 times more frequent than in blood [50,52]. In these anatomical sites, virus can persist in activated, replicating cells, as well as long-lived, latently infected cells, such as dendritic cells, macrophages and astrocytes. These sites may also have unique barriers to entry of cART, which limit the penetration of drugs.

### What type of cure might ultimately be achievable?

There are two potential strategies for cure. The first is what we might consider an "infectious diseases model" of cure, where the pathogen is treated and it disappears all together. This would require the elimination of all HIV-infected cells and for patients to have an HIV RNA count of less than 1 copy/ml. This is now commonly referred to as a sterilizing cure. The alternative approach would be to aim for remission or what we might consider a "cancer model" of cure, where an individual would have long-term health in the absence of treatment, with perhaps low-level viremia at less than 50 copies/ml. This is commonly referred to as a functional cure (Table 1).

**Table 1:** Overall potential strategies for curing HIV

**Sterilizing cure**	**Functional cure**
	
Infectious diseases model	Cancer model
Cure	Remission
Elimination of all HIV-infected cells	Long-term health in the absence of cART
HIV RNA <1 copy/ml	HIV RNA <50 copies/ml

cART, combination antiretroviral therapy

There are examples of both a sterilizing and functional cure that we need to learn from when designing new strategies for curing HIV. The recent case report of a German patient with acute myeloid leukemia, who received a bone marrow transplant from a donor who was resistant to HIV, is the only current example of a sterilizing cure [53]. The bone marrow donor carried a mutation in the CCR5 gene, a 32-base pair deletion, which knocks out expression of CCR5, the major coreceptor for HIV. Following transplantation, the patient stopped cART due to interactions with his chemotherapeutic drugs. Interestingly, virus did not rebound in the blood of this patient, and in more detailed studies, including multiple biopsies of his gastrointestinal tract, analysis of his cerebrospinal fluid (CSF) and lymph nodes, there were no detectable signs of HIV. The patient is now more than three years post transplant and HIV is still not detected. While a strategy of using bone marrow transplantation with a CCR5 mutant donor is not a realistic cure for HIV given the toxicity of the treatment, we need to comprehensively study this patient to fully understand how and why HIV was eliminated.

Elite controllers are another group that will teach us a lot about trying to achieve a functional cure. Elite controllers represent a unique group of patients who are able to achieve a consistent HIV RNA of less than 50 copies/ml in the absence of treatment [54]. There have been multiple studies examining the role of genetics, the virus and the immune response in elite controllers [55-57]. A consistent result from this work is the persistence of a robust HIV-specific T cell response in elite controllers, providing supportive evidence that inducing an effective immune response, perhaps via vaccination, may be a strategy to achieving a functional cure.

However, to date, the use of therapeutic vaccination in patients receiving cART has not been successful [58]. It is also important to note that approximately 7% of elite controllers experience a decline in their CD4+ T cells despite maintaining a viral load of less than 50 copies/ml. Ongoing virus replication and evolution, in addition to enhanced immune activation, has also been observed in these patients [55,59].

### Future and current strategies for cure

#### Treatment intensification

There have been a number of studies that have looked at the effect of treatment intensification on residual virus in patients receiving cART. These studies have included the addition of agents, such as Enfuvirtide, additional protease inhibitors or Raltegravir, to an already suppressive regimen [60-63]. Disappointingly, none of these studies have demonstrated any decline in low-level viremia or cell-associated HIV DNA. In addition, recently two small, non-randomized studies showed that treatment intensification had no significant effect on residual virus infection in the gastrointestinal tract (n = 7) [63] or in the cerebrospinal fluid (n = 10) [64]. Larger, randomized studies with longer follow up are still required to determine if treatment intensification may have any impact on persistent virus infection.

In one study, patients were randomized to Raltegravir intensification or to continue their current suppressive cART regimen. The addition of Raltegravir led to an increase in 2LTR circles within two weeks in one-third of patients, consistent with evidence of residual viral replication, although there was still no change in persistent low-level HIV RNA or cell-associated DNA, following intensification [62]. Therefore, although this study did not show an impact on the latent reservoir, the presence of active virus replication in some patients has significant implications for designing studies that may promote virus replication from latently infected cells.

#### Early treatment

Early treatment may be a potential strategy to reduce or even control the number of persistent latently infected cells. Several groups have demonstrated that the number of infected cells, as measured by cell-associated HIV DNA, decreases to a significantly lower level if treatment is initiated during acute rather than chronic infection [65,66]. Additionally, a recent longitudinal study demonstrated that in five of 32 (16%) patients who initiated treatment during acute infection, a viral load of less than 50 copies/ml was maintained after stopping cART (median of 77 months) [66].

However, this study was in contrast to many other reports of viral rebound in nearly all patients following cessation of cART, even when initiated during acute infection [67,68]. Why some but not all patients are able to control infection following treatment during acute infection is unclear. The role of very early treatment initiation in limiting seeding of the HIV reservoir, as well as preserving the immune responses capable of controlling HIV replication, requires further investigation.

#### Elimination of latently infected T cells

One strategy to eliminate latently infected cells is to convert these cells into activated cells. Activation of latently infected T cells would induce virus production and subsequent cell death, while further rounds of infection would be blocked by cART. IL-7 is a cytokine that can effectively do this in the laboratory [69]. IL-7 has also recently been shown to be safe and well tolerated in patients with HIV infection [70,71]. One concern, however, with IL-7 is that this cytokine may also induce the proliferation of latently infected cells without activating them [27]. IL-7 is currently undergoing clinical trials (ERAMUNE, http://www.clinicaltrials.gov), as a strategy to reduce the size of the latent reservoir, and results of this trial are awaited with high interest.

There are alternative compounds, such as prostratin, that can promote T cell activation and HIV transcription *in vitro *[72]. However, prostratin has not yet been trialled in any human studies.

Alternatively, a more targeted approach would be to turn on the HIV genes within the latently infected cells. In a latently infected cell, the HIV genes are silent and turned off. Histone deacetylase inhibitors (HDACi) are drugs that can modify gene expression by changing the acetylation state of genes. These drugs are also able to turn HIV genes on in latently infected cells *in vitro*. In cancer cells, HDACi induce cell death of the malignant cells and many HDACi are now in advanced clinical development for the treatment of different cancers [73,74]. Although valproic acid, a relatively weak HDACi, showed promising effects in a small pilot study [75], further retrospective studies failed to demonstrate any benefit from this intervention [76-78].

A far more potent HDACi, Vorinostat (also called SAHA), is already licensed for the treatment of cutaneous T cell lymphoma, is well tolerated in humans, and has significant activity in promoting HIV or turning HIV genes on *in vitro *[79,80]. Other drugs, such as methylation inhibitors, have a similar effect in promoting HIV transcription in latently infected cells. The most potent effect observed in laboratory models, however, results when a combination of drugs is used [72,81]. It is therefore likely that the elimination of latently infected cells *in vivo *will require the addition of more than a single drug to a patient's cART regimen.

None of these strategies, however, specifically target HIV-infected cells, and latently infected cells are rare. On average, they occur one in a million, or one in a 100,000 cells [18]. Therefore, these current strategies could potentially have effects on uninfected cells leading to toxicities and, therefore, the risk benefit of these strategies needs to be carefully evaluated.

#### Making cells resistant to HIV

Future strategies aimed at making CD4+ T cells resistant to HIV are also currently being investigated, which would ultimately allow for the cessation of cART. Some approaches have included gene therapy to reduce expression of the chemokine receptor CCR5. This has been successfully performed in mice through the introduction of a zinc finger nuclease, which inhibits CCR5 expression, into the CD34+ hemapoietic progenitor cells. This led to a reduction in the expression of CCR5, and following HIV infection of these mice, CD4+ T cells did not decline [82].

An alternative approach is to use RNA-based gene therapy to reduce CCR5 expression, as well as specifically inhibit HIV replication [83]. This approach was recently tested in four HIV-infected patients with AIDS-associated lymphoma, who received a transplant with three RNA-based gene products as part of the transplant. The investigators demonstrated that this procedure was safe and that the transferred genes persisted in a subset of cells for 24 months. Although widespread use of these therapies is many years away, these results are encouraging for the possible development of a gene therapy-based treatment strategy that may achieve a functional cure.

### What are the main priorities now?

First, universal access to cART still remains the major priority for the management of patients with HIV. cART will always be a part of any strategy that may lead to a cure. Second, there is an urgent need for clinical trials. There are several compounds that look promising in the laboratory, including vorinostat and IL-7. It is highly likely that a combination of approaches will be needed together with cART intensification. These studies are likely to have the greatest possibility of success in patients who initiated cART shortly after acute infection.

Importantly, more active community engagement in this work is critical. Basic science issues are often perceived as highly technical and without impact on the daily lives of infected or affected communities. It is, however, crucial for community representatives and basic science researchers to work together to systematically address the barriers and challenges that hold us back from finding a cure.

Clinical trials will be needed to move the field forward and it is essential that affected communities are involved in these efforts as true partners. For example, it is important that community representatives are involved in longer term strategic planning for eradication studies, as well as the planning of individual studies. Community members should be invited to join the steering committees, advisory boards, and data safety and monitoring boards of these studies. Additionally, they should join together with health professionals in raising the awareness and understanding of issues related to HIV persistence and potential eradication. Such an alliance will also be critical for increasing the funding support for basic science research in the field of HIV.

As we move forward into clinical trials, we also need to carefully consider what the most appropriate endpoints should be. Can we use surrogate markers of the reservoir, including HIV DNA and plasma viremia? Are there circumstances in which it will be acceptable to trial treatment interruption with the well-documented risks of viral rebound [84]?

## Conclusions

We should not continue to accept that HIV is a long-term chronic illness that commits patients to life-long treatment and associated toxicities. We should not accept that life-long treatment may not be available to all who need it. A cure will need a great scientific advance, but we will not achieve a cure with science alone. We need scientists, clinicians, affected communities, industry, politicians and government to embrace the challenge and work together towards finding a cure for HIV.

## Competing interests

The authors declare that they have no competing interests.

## Authors' contributions

SRL wrote the manuscript. VAE prepared the figure and table and contributed to the critical revision of the manuscript. JHE, BS and NC reviewed the manuscript and provided helpful comments. All authors read and approved the final manuscript.
